# Orbitofrontal neurons signal reward predictions, not reward prediction errors

**DOI:** 10.1016/j.nlm.2018.01.013

**Published:** 2018-01-31

**Authors:** Thomas A. Stalnaker, Tzu-Lan Liu, Yuji K. Takahashi, Geoffrey Schoenbaum

**Affiliations:** a National Institute on Drug Abuse Intramural Research Program, Cellular Neurobiology Research Branch, Behavioral Neurophysiology Research Section, 251 Bayview Blvd., Baltimore, MD 21224, United States; b Department of Anatomy and Neurobiology, University of Maryland School of Medicine, 20 Penn St., Baltimore, MD 21201, United States; c Solomon H. Snyder Department of Neuroscience, The Johns Hopkins University, Baltimore, MD 21218, United States

**Keywords:** Orbitofrontal, Learning, Reward prediction error, Single unit, Rat

## Abstract

Neurons in the orbitofrontal cortex (OFC) fire in anticipation of and during rewards. Such firing has been suggested to encode reward predictions and to account in some way for the role of this area in adaptive behavior and learning. However, it has also been reported that neural activity in OFC reflects reward prediction errors, which might drive learning directly. Here we tested this question by analyzing the firing of OFC neurons recorded in an odor discrimination task in which rats were trained to sample odor cues and respond left or right on each trial for reward. Neurons were recorded across blocks of trials in which we switched either the number or the flavor of the reward delivered in each well. Previously we have described how neurons in this dataset fired to the predictive cues (Stalnaker et al., 2014); here we focused on the firing in anticipation of and just after delivery of each drop of reward, looking specifically for differences in firing based on whether the reward number or flavor was unexpected or expected. Unlike dopamine neurons recorded in this setting, which exhibited phasic error-like responses after surprising changes in either reward number or reward flavor (Takahashi et al., 2017), OFC neurons showed no such error correlates and instead fired in a way that reflected reward predictions.

## 1. Introduction

Neurons in the orbitofrontal cortex (OFC) fire in anticipation of and during biologically significant events (Rolls, 1996; Wallis, 2012). In the case of rewards, such firing has been characterized as reflecting value or some combination of the value and other features of the rewards themselves (Blanchard, Hayden, & Bromberg-Martin, 2015; Padoa-Schioppa & Assad, 2006; Stalnaker et al., 2014; Thorpe, Rolls, & Maddison, 1983). Anticipatory firing that predicts information about expected rewards is generally thought to explain, in some manner, why the OFC is often necessary for adaptive behavior that is based on knowledge of the specific rewards to be delivered (Rudebeck & Murray, 2014; Stalnaker, Cooch, & Schoenbaum, 2015; Wallis, 2012). In addition, the role for OFC in signaling reward predictions could explain why this area can, under some circumstances, be necessary for learning (Schoenbaum, Roesch, Stalnaker, & Takahashi, 2009). That is, predictions broadcast by OFC neurons could be utilized by downstream areas calculating reward prediction errors. Consistent with this, we have shown that OFC lesions result in a diminution of expectancy-related changes in the firing of midbrain dopamine (DA) neurons, as if input critical to calculating accurately the underlying predictions had been lost or degraded (Takahashi et al., 2011). This idea stands in contrast to a simpler proposal, supported by some single unit and fMRI data, that OFC directly signals reward prediction errors (Knutson & Wimmer, 2007; Nobre, Coull, Frith, & Mesulam, 1999; O'Doherty, Dayan, Friston, Critchley, & Dolan, 2003; Sul, Kim, Huh, Lee, & Jung, 2010; Thorpe et al., 1983; Tobler, O'Doherty, Dolan, & Schultz, 2006).

So, do OFC neurons signal reward predictions errors or do they signal reward predictions? Here we tested this question directly by analyzing the firing of OFC neurons recorded in an odor discrimination task in which rats were trained to sample odor cues and respond left or right on each trial for reward. Neurons were recorded across blocks of trials in which we switched either the number or the flavor of the reward delivered in each well. Previously we have described how neurons in this dataset fired to the predictive cues (Stalnaker et al., 2014); here we focused on the firing in anticipation of and just after delivery of each drop of reward, looking specifically for differences in firing based up on whether the reward number or flavor was unexpected or as expected. Unlike DA neurons recorded in this setting, which exhibited phasic error-like responses after surprising changes in either reward number or reward flavor (Takahashi et al., 2017), OFC neurons showed no such error correlates and instead fired in a way that reflected reward predictions.

## 2. Material and methods

### 2.1 Subjects

Male Long-Evans rats were obtained at 175–200 g (approximately 60 days old on arrival) from Charles River Labs, Wilmington, MA. Rats were tested at the University of Maryland School of Medicine in accordance with School of Medicine and NIH guidelines.

### 2.2 Surgical procedures and histology

Surgical procedures followed guidelines for aseptic technique. Electrodes, consisting of drivable bundles of eight 25-um diameter FeNiCr wires (Stablohm 675, California Fine Wire, Grover Beach, CA) electroplated with platinum to an impedance of ~ 300 kΩ, were manufactured and implanted as in prior recording experiments. Driveable electrodes were implanted in the left orbitofrontal cortex of six rats (3.0 mm anterior to bregma, 3.2 mm laterally, and, to begin, 4.0 mm ventral to the surface of the brain) in each rat. At the end of the study, the final electrode position was marked, the rats were euthanized with an overdose of isoflurane and perfused, and the brains were removed from the skulls and processed using standard techniques.

### 2.3. Behavioral task

**Fig. 1.**
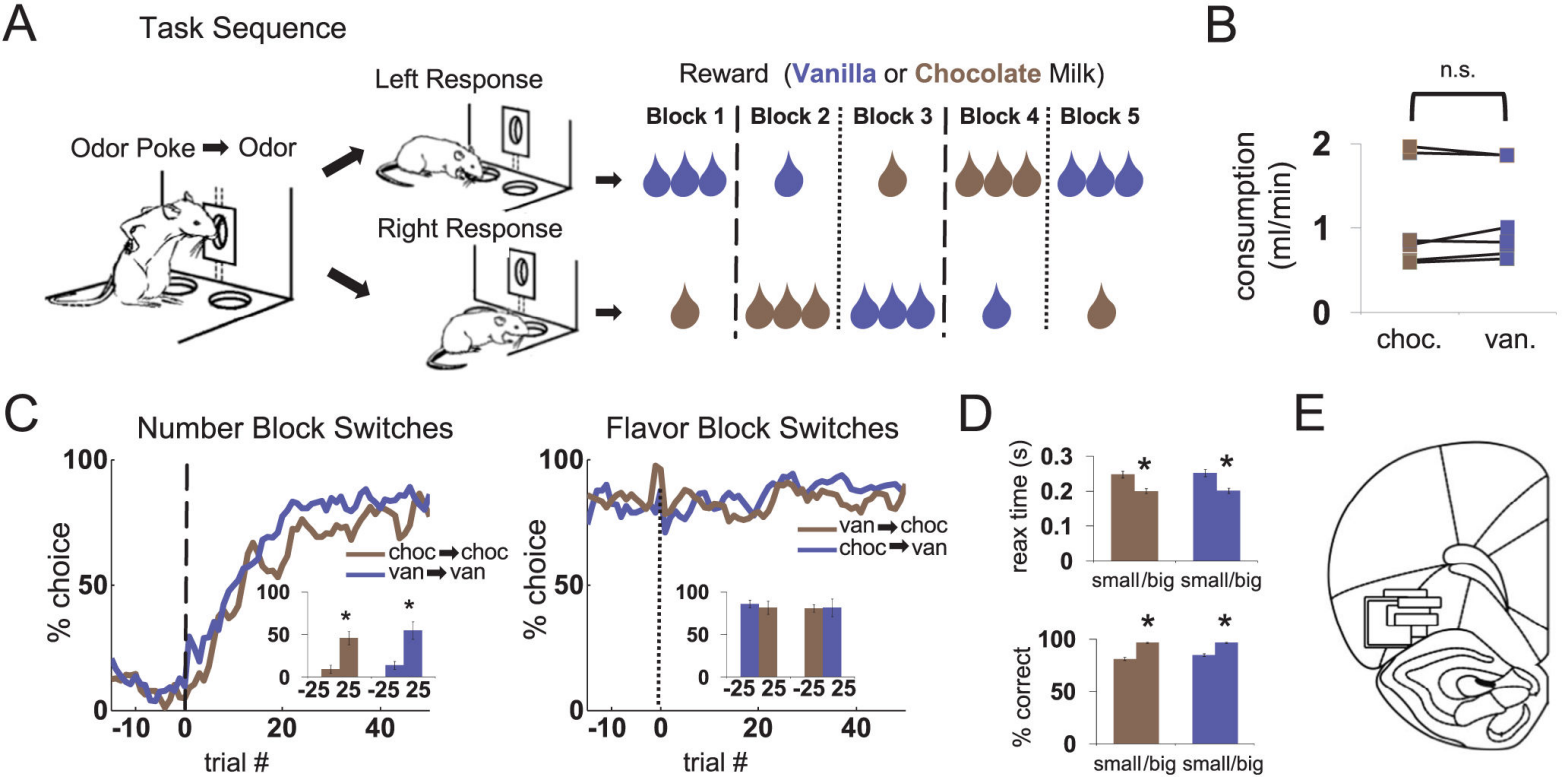
Behavior and Histology. A. Task sequence. After initiating a trial with a nosepoke, an odor was delivered for 500 ms, after which rats responded at one of the two fluid wells for 1 or 3 drops of chocolate or vanilla milk solution, delivered 500 ms after the well poke. Two odors indicated forced choices, left or right; a third odor indicated free choice. Reward contingencies were stable across blocks of ~ 60 trials, but switched in number of drops (dashed lines) or flavor (dotted lines) in four unsignaled transitions. Rewards in the two directions always differed in both number of drops and flavor (only one of the four possible block sequences is shown). B. Chocolate and vanilla milk were equally preferred in a ten minute consumption test in a separate group of rats (t_10_ = 0.1, p = .93). C. Free-choice rates in the task reflected the number of drops but not the flavor. Number block switches (left panel) had a similarly large effect on choice rates for chocolate → chocolate compared to vanilla → vanilla switches. Flavor block switches (right panel) had no effect on choice rates for big vanilla → big chocolate or big chocolate → big vanillla switches. Line figures show average trial-by-trial choice rates on free-choice trials on an x-axis scale that includes all interleaved correct free- and forced-choice trials; inset bar graphs compare average choice rate on all free-choice trials within the last 25 before a block switch and the first 25 after a block switch (again, this 25-trial period includes interleaved correct free- and forced- choice trials). ANOVA on difference in choice rates across transitions, with factors transition type and initial flavor; main effect of transition type (F_1,92_ = 195.7, p < .001), driven by significant changes across number transitions (planned contrast, F_1,92_ = 445.9, p < .0001), and insignificant changes across flavor transitions (planned contrast, F_1,92_ = 1.3, p = .27); no effect of initial flavor (F_1,92_ = 0.0, p = .93); no differences between vanilla-to-chocolate and chocolate-to-vanilla (planned contrast. F_1,92_ = 2.3, p = .13). A focused comparison of the magnitude of changes across number and flavor transitions included in the subsequent analyses, between this experiment and a separate one in which dopamine neurons were recorded revealed the following results: No difference across included flavor transitions (t_137_ = 0.6, p = .57) and larger changes in choice rate across number transitions in this experiment than in the dopamine experiment (t_249_ = 3.1, p < .01). D. Reaction time (top panel) and accuracy (bottom panel) reflected the number of drops expected but not the flavor. Bar graphs show average reaction time (from end of odor to start of movement) or accuracy on forced-choice trials within the last 25 trials of blocks. Within-subjects ANOVAs on reaction time and accuracy: main effects of reward number (F_1,93_ = 62.2, p < .001; F_1,93_ = 182.3, p < .001) but not flavor (F_1,93_ = 0.3, p = .57.; F_1,93_ = 5.3, p = 0.024^†^), nor interactions (F_1,93_ = 0.1, p = .73.; F_1,93_ = 5.1, p = 0.027^†^). Two additional ANOVAs on reaction time and accuracy compared this experiment and a separate one in which dopamine neurons were recorded. These revealed no interactions of flavor or reward number with experiment (reward number: F_1,124_ = 0.5, p = .49; F_1,124_ = 1.6, p = .20; flavor: F_1,124_ = 0.0, p = .98; F_1,124_ = 0.0, p = .96). E. Recording sites in OFC. The black boxes indicate the approximate location from which recordings were made in each rat (in the left hemisphere). The width represents the estimated span of the electrode bundle (— 1 mm), and the height represents the approximate extent of recording across all sessions. Bregma + 2.8 to 3.6 mm. ^†^Not significant if corrected p-value criterion is used (p < .0167 by Bonferroni correction), so that the family- wise p-criterion across the three separate ANOVAs on flavor, in panels C–D, was equal to 0.05.

Recording was conducted in aluminum chambers, on one wall of which was a panel with an odor port and two fluid wells arranged below it (see Fig. 1). The odor port was connected to an air flow dilution olfactometer to allow the rapid delivery of olfactory cues. The fluid wells were connected to fluid delivery lines containing flavored milk (Nesquick brand chocolate or vanilla) diluted 50% with water. Delivery of odors at the odor port and the fluids at the fluid wells was controlled by a custom C+ + program interfaced with solenoid valves. Photobeam breaks at the port and wells were monitored and recorded by the program. A houselight was also controlled by the program.

Rats were trained extensively before they were implanted with electrodes. After implantation, we retrained rats to work while attached to the recording cable. Each training session included as many trials as a rat would perform before quitting, ~150–250. This initial shaping phase gradually introduced all elements of the task (described below), and thus rats could learn the associative structure of the task over this period. Recording was begun when rats could complete five blocks of trials (at least 260 trials) with the cable. Total number of pre-recording training sessions averaged 32.5 (ranging from 24 to 43).

Each recording session consisted of a series of self-paced trials organized into five blocks. Rats could initiate a trial by poking into the odor port while the house light was illuminated. Beginning 500 ms after the odor poke, an odor would be delivered for 500 ms. If the rat withdrew from the odor port before completion of the 1000 ms preodor + odor period, the trial would be aborted and the houselight turned off. The end of the odor served as a go-response indicating that rats could respond by moving from the odor port to the left fluid well or the right fluid well, after which they had to wait for 500 ms before fluid delivery began. The identity of the odor specified whether they could receive reward at the left well (forced-choice left), the right well (forced-choice right), or either well (free-choice). The identity and meaning of these odors remained the same across the entire experiment. Odors were presented in a pseudorandom sequence such that the free-choice odor was presented on 7/20 trials and the left/right odors were presented in equal numbers (± 1 over 250 trials). In addition, the same odor could be presented on no more than 3 consecutive trials.

Rewards were either one drop or three drops of chocolate or vanilla milk, with drop size ~0.05 ml and 500 ms between drops. Response-reward contingencies were consistent within blocks of trials, such that the same reward would be delivered for every correct right response, either free- or forced-choice, and a different reward would be delivered for every correct left response, free- or forced-choice. Upon each block transition, either the number of drops would change on both sides (1 drop to 3 drops and vice versa) and flavor remained constant, or the flavor would change on both sides (chocolate to vanilla and vice versa) and the number of drops remained constant. These block transitions were not explicitly signaled and could not be predicted based on the exact number of trials. The first block, consisting of on average 43 ± 16 (SD) trials, was used to set the rats’ expectations before the first transition. The length of the last four blocks varied non-systematically around 65 ± 11 (SD). The reward schedule was arranged so that in each block, reward features available on one side were always paired with the opposite reward features on the other side – thus when one drop of chocolate milk was available on the left, three drops of vanilla was available on the right, etc., resulting in a total of four different reward combinations. The reward combination in the first block was randomly chosen, after which the block order followed a set scheme consisting of a drop-number transition, a flavor transition, another drop-number transition, and another flavor transition (see Fig. 1A for an illustration of one of the four possible block schedules).

During testing, rats were limited to 10 min of *ad lib* water each day, in addition to fluid earned in the task.

### 2.4 Flavor preference testing

In six rats from a separate experiment (same strain and source, and same water restriction regimen), we compared consumption of the chocolate vs. vanilla milk solution in two-bottle tests. All rats were tested for ten total minutes, with the location of the bottles swapped every 30 s. Two rats were given five 2-min tests while the other four rats were given one 10-min test each.

### 2.5 Single-unit recording

Procedures were the same as described previously (Stalnaker, Calhoon, Ogawa, Roesch, & Schoenbaum, 2010). Wires were screened for activity daily; if no activity was detected, the rat was removed and the electrode assembly was advanced 40 or 80 um. Otherwise a session was conducted, and the electrode was advanced by at least 40 um at the end of the session. Neural activity was recorded using Plexon Multichannel Acquisition Processor systems (Dallas, TX), interfaced with odor discrimination training chambers. Signals from the electrode wires were amplified and filtered by standard procedures described in previous studies. Waveforms (> 2.5:1 signal-to-noise) were extracted from active channels and recorded with event timestamps sent by the behavioral program.

### 2.6 Data analysis

Units were sorted using Offline Sorter software from Plexon Inc. (Dallas, TX), using a template matching algorithm. Sorted files were then processed in Neuroexplorer to extract unit timestamps and relevant event markers. These data were subsequently analyzed in Matlab (Natick, MA).

We first screened neurons by whether they showed a significant increase in firing rate in from 100 to 500 ms after delivery of the first drop of reward compared to baseline, defined as the last two seconds of the preceding inter-trial interval (by *t*-test across all trials, p < .05). We designed the subsequent analyses using two different epochs that surrounded the timepoint of each drop delivery (the timestamp at which the solenoid was activated to begin reward delivery) or omission (the timepoint at which solenoid activation would have been expected, 500 ms after the previous reward delivery timestamp). The reward response epoch was from 100 ms to 300 ms after the specified reward timepoint. The complementary reward anticipation epoch began 200 ms before the reward timepoint and ended 100 ms after it. For each analysis, we calculated a prediction error or prediction score, defined as the difference between the average firing rate in the early trials of the block in question and that in the late trials of the same block or the previous block, as appropriate. We then performed t-tests on the distribution of scores across all included neurons (p < .05). For the analyses of flavor shifts presented in Figs. 3 and 4, we first separated neurons recorded across flavor shifts that resulted in significant variability in established behavior from neurons recorded across shifts that did not. This was done to rule out a lack of error signaling due to a failure to attend to flavor, since the two flavors were so similar and the rats so experienced that it was not obvious whether they had even noticed the shift in flavor. A shift with significant behavioral effect was defined as one in which at least one of the following four conditions was true: (a) one of the first two free-choices after the shift was towards the 1-drop side; (b) the number of licks was significantly different in the last ten vs. first ten trials on either side (by *t*-test, p < .05); (c) the error rate on forced-choice trials was significantly different on the last ten vs first ten trials on either side (by the binomial test, p < .05); (d) the response latency was significantly different in the last ten vs. first ten forced-choice trials on either side (by *t*-test, p < .05). For these flavor change analyses, scores were calculated for each neuron on each shift. For all figures, population averaged activity was baseline subtracted on each trial before averaging.

We also ran an ANOVA on firing rate immediately after reward on rewarded trials (on the 3-drop side from 100 ms after the first drop to 300 ms after the second drop; on the 1-drop side, from 100 to 300 ms after the first drop). This ANOVA had factors side (i.e. direction), number of drops, and flavor. We then analyzed neurons with a main effect of flavor by comparing the magnitude of changes across flavor shifts (last five trials of previous block vs. first five of new block) with those across the block after flavor shifts (first five trials of the new block vs. the last five trials of that block).

### 2.7 Dopamine neurons

**Fig. 2.**
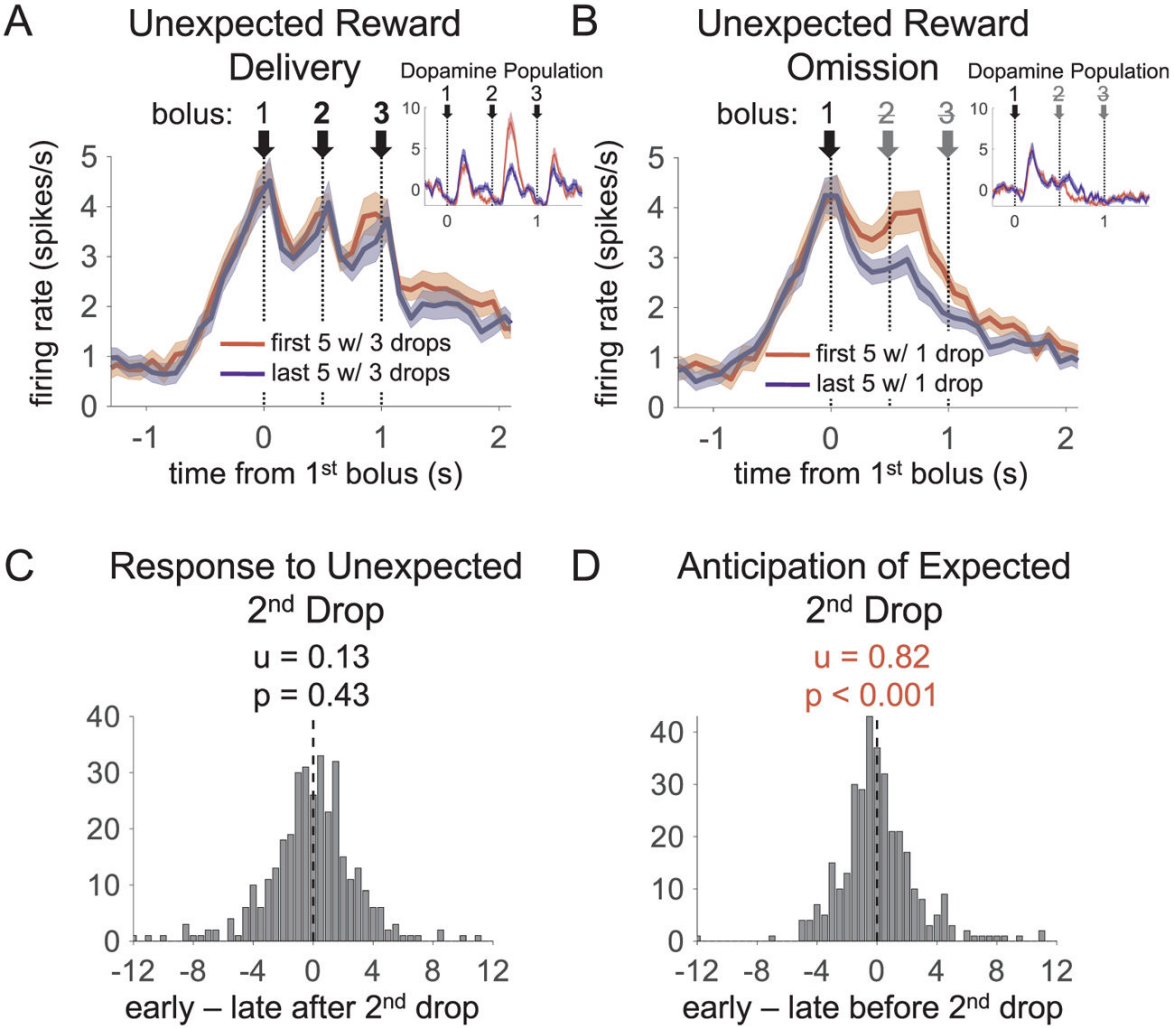
Reward-evoked activity of reward-responsive orbitofrontal cortical neurons (n = 347) after shifts in reward number. (A and B) Average baseline-subtracted firing on first five (red) and last five (blue) trials after a shift in reward number, from one drop to three drops (A) and from three drops to one drop (B). Both correct free- and forced-choice trials were included. Shading represents the standard error at each bin. (C and D) Distribution of difference scores for the epoch from 100 to 300 ms after the unexpected second drop (C), in which dopamine neurons reflect a positive prediction error (inset in A), and for the epoch from 200 ms before to 100 ms after the time of the omitted second drop (D), which precedes the dopamine negative prediction error response (inset in B). OFC neurons thus fail to signal prediction errors (A and C) but do signal outcome predictions (B and D). Statistics above histograms show average difference score and p-value for a *t*-test on the population (for C, t_346_ = 0.79; for D, t_346_ = 3.7). The dopamine population had significantly higher positive prediction error indices than those of the OFC population shown in C (t_405_ = 5.1, p < .0001) and the OFC population had significantly higher anticipatory indices, shown in D, than those in the dopamine population (t_405_ = 2.9, p < .01). (For interpretation of the references to colour in this figure legend, the reader is referred to the web version of this article.)

**Fig. 3.**
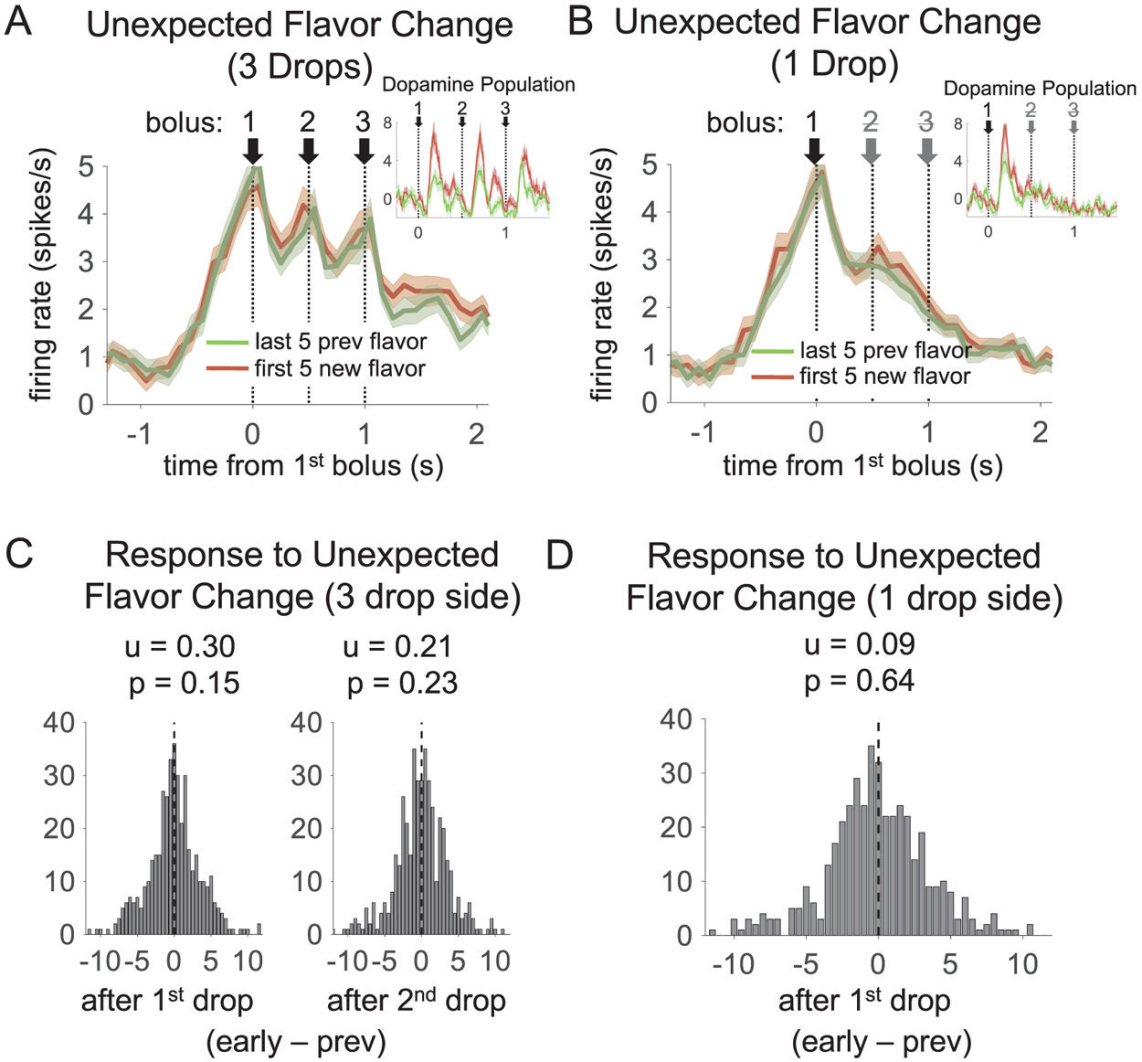
Reward-evoked activity of reward-responsive orbitofrontal cortical neurons after shifts in reward flavor. (A and B) Average baseline- subtracted firing on first five trials after a shift in reward flavor (red) versus last five trials from the previous block (green), on the 3-drop side (A) and on the 1-drop side (B). Both correct free- and forced-choice trials were included. The number of drops on each side remained constant across the shift. Shading represents the standard error at each bin. (C-D) Distribution of difference scores for the epochs from 100 to 300 ms after the first and second drops of the new flavor on the 3-drop side (C), and after the first (and only) drop of the new flavor on the 1-drop side (D). Dopamine neurons showed a significantly positive prediction error score in response to flavor changes at each of these timepoints (insets in A and B), whereas the OFC population did not. Statistics above the histograms show average difference score and p- value for a *t*-test on the population, with each neuron X shift providing a datapoint (For C, t_431_ = 1.5, left panel, t_346_ = 1.2 for right panel; for D, t_431_ = 0.47). Flavor shifts were only included when behavior showed evidence of the shift (104 of 176 total flavor shifts, on which were recorded 296 neurons; see Section 2 for definition of behavioral evidence of a shift). The dopamine population had a significantly higher prediction error score than that in the OFC population for each bolus of new flavor (first drop on 3-drop side: t_478_ = 2.1, p < .05; second drop on 3-drop side: t_478_ = 2.4, p < .05; first drop on 1- drop side: t_478_ = 2.1, p < .05). (For interpretation of the references to colour in this figure legend, the reader is referred to the web version of this article.)

We compared these data with recordings from dopamine neurons recorded in rats in the same task, as reported earlier (Takahashi et al., 2017). Figures from that dataset (insets in Figs. 2 and 3) are slightly modified from those shown in the previous paper (error shading, lines indicating drops of reward, and the x-axis scaled are modified). The statistical comparison between OFC and dopamine neuronal responses used dopamine indices as calculated in that paper.

## 3. Results

We recorded single-unit activity from OFC as rats performed a task with changing amounts and flavors of a milk solution reward (see Fig. 1 for task, behavior, and histology; all statistics are listed in figure legends). Because we sought to compare OFC neural activity with that dopamine neuronal recordings done in the same task and reported separately, we also compared behavior between the two experiments (summarized in the Fig. 1 figure legend, C and D). For all such comparisons, there was no difference except that in the OFC dataset there was a slightly greater effect of changes in the number of reward drops on choice rate. We focused our neural analysis on the reward-responsive population, consisting of neurons with a significant increase in firing to the first drop of reward relative to baseline, including both free- and forced-choice trials (this first drop of reward was delivered on all trials). Of the 831 total OFC neurons, 347 (42%) recorded across 88 sessions were reward-responsive by this criterion. All analyses reported below examined this subpopulation, but we saw similar results across the entire OFC population. All analyses reported below included all correct free- and forced-choice trials.

At a first glance, the response of OFC population to reward (see Fig. 2A and B) was almost the inverse of that of DA neurons recorded in the same task (Takahashi et al., 2017). In our hands in this task, DA neurons begin to respond about 100 ms after reward delivery and peak 200–300 ms after each reward drop, whereas the OFC neurons began to respond before each drop of reward, peaked about 100 ms after delivery, and reached a local minimum 250–300 ms after reward delivery. These contrasting patterns are qualitatively inconsistent with the proposal that OFC neurons respond to prediction errors and are more in accord with activity in anticipation of reward.

To test directly whether OFC neurons signaled prediction errors, we examined OFC activity in an epoch from 100 to 300 ms after delivery or omission of the second drop of reward at the beginning versus the end of blocks 2 and 4 in our task. In these blocks, the reward shifts from 1 drop to 3 drops on one side, and 3 drops to 1 drop on other side, with the flavor remaining constant. Thus, prediction errors occur after the newly delivered second drop on the 3-drop side, and after the newly omitted second drop on the 1-drop side. These errors are strongly reflected in the firing of DA neurons (Takahashi et al., 2017). By contrast, the population of reward-responsive OFC neurons did not fire differently after the second drop when it was unexpected versus when it had become expected at the end of that same block (see Fig. 2A and C). OFC neurons did show an increased firing rate after the omission timepoint. However, this increase began *before* the time of expected reward (see Fig. 2B and D) and even on the initial trial of the omission (data not shown). In both the response after the unexpected second drop and in the anticipatory response to the absent second drop, the OFC population response was significantly different than the dopamine population response (statistical analyses detailed in the figure legend). Thus the features of OFC activity are consistent with a prediction of the expected second drop, rather than a prediction error in response to its omission.

A similar pattern in which OFC neurons signaled reward predictions rather than prediction errors was evident when reward flavor changed unexpectedly. At the start of blocks 3 and 5, 3 drops of chocolate milk solution replaced 3 drops of vanilla milk solution (or vice versa) on one side, and 1 drop of chocolate replaced 1 drop of vanilla milk solution (or vice versa) on the other side. Although the two flavors were equally preferred, DA neurons still showed a robust phasic increase after this shift, which declined with learning in the block like a prediction error (Takahashi et al., 2017). This increase was most prominent immediately after the first and second drops on the 3-drop side, and immediately after the first drop on the 1-drop side. While there may be several interpretations of the information carried by this change in firing, the most parsimonious one is that DA neuron firing registers a prediction error based on the change in reward flavor. Again, the OFC reward-responsive population showed the inverse pattern of response: it increased in anticipation of each drop of reward, but as a population it showed no change after each drop of the unexpected flavor (Fig. 3A–D). A direct statistical comparison revealed that the DA population had a significantly greater response than the OFC population at each of the three time points taken separately (see Fig. 3 legend). Note that this lack of OFC prediction error signaling after flavor shifts does not mean that specific OFC neurons failed to track changes in flavor. Indeed, many OFC neurons showed effects of flavor in the same time epoch in which we examined error signaling and therefore tended to change their firing in the first few trials after flavor shifts (144 of 347 total reward-responsive neurons showed a main effect of flavor or an interaction of flavor with direction or number of drops; see Section 2); however, these subpopulations did not show greater signaling when their preferred flavor was unexpected vs. when it was expected and thus failed to conform to prediction error signals. For example, although by definition the firing rates of neurons with a main effect of flavor by ANOVA across all trials (n = 53) changed significantly across flavor shifts (after second drop on 3-drop side, t_52_ = 3.2, p < .01; after first drop on 1-drop side, t_52_ = 4.8, p < .0001), they were no different immediately after flavor shifts vs. the end of those same blocks (after second drop on 3-drop side, t_52_ = 0.4, p = .71; after first drop on 1-drop side, t_52_ = 0.03, p = .97).

**Fig. 4.**
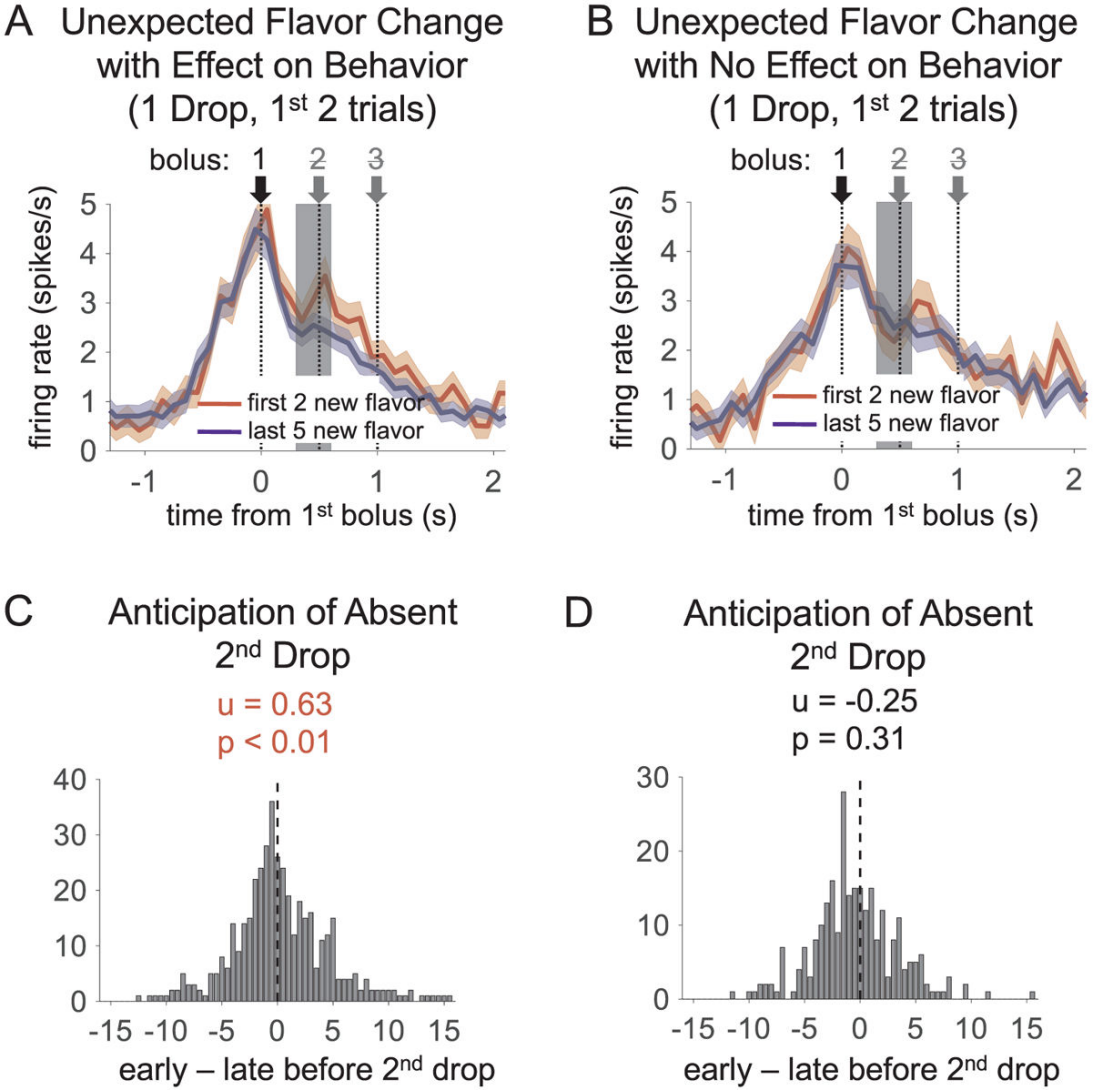
The OFC response in anticipation of the absent second drop after a flavor shift, comparing the response when the rat showed behavioral evidence of the shift (left) to that when the rat showed no evidence of the shift (right). (A and B) Average baseline-subtracted firing on first two trials after a shift in reward flavor (red) versus last five trials from that block (blue), on the 1-drop side when behavior reflected the change (A) and when it did not (B). Both correct free- and forced-choice trials were included. Colored shading represents standard error at each bin. The phasic increase in A early in the block in the gray-shaded epoch shows that the OFC population made a reward prediction based on the flavor change, even though that change did not elicit a prediction error signal in this population (see main text). (C and D) Distribution of difference scores for the epoch from 200 ms before the time that the second drop would be expected, to 100 ms after it. Statistics above the histograms show average difference score and p-value for a *t*-test on the population, with each neuron X shift providing a datapoint (For C, t_431_ = 2.7; for D, t_261_ = −1.0; for the comparison between C and D, t_692_ = − 2.5, p = .014). See Section 2 for definition of behavioral evidence of a flavor shift. (For interpretation of the references to colour in this figure legend, the reader is referred to the web version of this article.)

In contrast to the lack of prediction error signaling after unexpected flavor changes, we still observed reward predictive signaling immediately after these shifts. This was evident in some blocks after the flavor shifts on the 1-drop side. Here, the flavor that had previously been delivered in three drops (e.g. chocolate or vanilla) was delivered as only a single drop. If rats were attending to flavor, that particular flavor might be seen as predicting two additional drops right after the shift. Indeed, we have previously reported that rats’ behavior can subtly reflect this prediction (Stalnaker et al., 2014). This is evident in some blocks as a transient increase in free-choice rate towards the one drop side for 1–2 trials immediately after the shift. Here we found that the activity in the OFC neurons recorded in blocks where behavior indicated the rats were attending to flavor (see Section 2) reflected this prediction (see Fig. 4A and C). OFC neurons recorded in these blocks showed a transient phasic increase immediately before the time of the expected second drop for the first two trials after the shift. Interestingly, this predictive activity was not present in OFC neurons recorded in blocks in which rats did not show evidence that they were attending to flavor (see Fig. 4B and D). In summary then, after both value-related and value-neutral changes in reward, OFC neural activity correlated with reward predictions rather than prediction errors.

## 4. Discussion

As noted at the outset, it has become widely accepted that the OFC is important for signaling information about expected rewards (Rudebeck & Murray, 2014; Stalnaker et al., 2015; Wallis, 2012). Such anticipatory activity is thought to explain the role this area plays in a variety of behaviors (Camille, Griffiths, Vo, Fellows, & Kable, 2011; Gallagher, McMahan, & Schoenbaum, 1999; Gourley et al., 2013; Izquierdo, Suda, & Murray, 2004; Jones et al., 2012; Ostlund & Balleine, 2007; Reber et al., 2017). However, the OFC is also often important for learning (Jones & Mishkin, 1972; McDannald, Lucantonio, Burke, Niv, & Schoenbaum, 2011; McDannald, Saddoris, Gallagher, & Holland, 2005; Takahashi et al., 2009; Tsuchida, Doll, & Fellows, 2010; Walton, Behrens, Buckley, Rudebeck, & Rushworth, 2010). While we have suggested that OFC-dependent reward predictions might also underlie the OFC’s role in learning (Schoenbaum et al., 2009), it is also possible that OFC might drive learning more directly by signaling mistakes or errors in reward prediction (Knutson & Wimmer, 2007; Nobre et al., 1999; O'Doherty et al., 2003; Sul et al., 2010; Thorpe et al., 1983; Tobler et al., 2006).

Here we addressed this question directly by analyzing single unit activity recorded in OFC in rats performing a task that we have previously used to identify reward prediction error correlates in midbrain DA neurons (Takahashi et al., 2017). We found that the firing of OFC neurons did not appear to correlate with errors in reward prediction and instead seemed to provide a complementary signal, anticipating expected rewards. This finding is consistent with several prior studies that have looked for and failed to see error signals in single units recorded in OFC (Kennerley, Behrens, & Wallis, 2011; McDannald et al., 2014; Takahashi et al., 2013, 2009). This study extends those prior reports by testing whether activity in OFC reflects errors in the prediction of the sensory features of the rewards, independent of value. Such identity or state prediction errors have been previously reported in BOLD signal in a variety of brain areas (Boorman et al., 2016; Glascher, Daw, Dayan, & O'Doherty, 2010), including the OFC and related structures. The current data indicates that these signals are not present in single unit activity recorded from OFC. Single unit recordings are likely to be biased to include mostly large pyramidal output neurons, thus this dichotomy suggests the OFC may be an important recipient of such signals but that it likely does not pass them along to downstream areas.

The complementary relationship identified here between activity in OFC and the DA neurons replicates prior findings in a variant of this task in which we manipulated reward value by changing either number or timing (Takahashi et al., 2009). In that setting, we also found firing in advance of expected rewards in OFC and firing after unexpected rewards in midbrain DA neurons. We have recently reported error-like signaling in DA neurons in response to changes in reward flavor in this task (Takahashi et al., 2017). Combined with this report, the current findings show that a similar complimentary relationship exists for value-neutral sensory information. This demonstration is significant for understanding OFC function, since it highlights the role of the OFC in predicting actual associative information about expected rewards, such as their timing, quantity, and quality, in addition or perhaps instead of simply representing their utility or common value. Previously we have shown that value-based dopaminergic prediction errors are disrupted by OFC lesions in a manner consistent with a role for OFC in shaping the underlying predictions (Takahashi et al., 2011). Based on the current data and other work implicating the OFC in learning about specific reward information (McDannald et al., 2011; McDannald et al., 2005; Ostlund & Balleine, 2007), we would predict that sensory prediction errors in DA neurons are equally or perhaps even more dependent on OFC.
